# THE VIRTUE OF A LIFE OF COMPLEXITY: THE KEY TO HEALTHY AGING

**DOI:** 10.1093/geroni/igz038.2230

**Published:** 2019-11-08

**Authors:** Lewis Lipsitz

**Affiliations:** Hebrew SeniorLife, Roslindale, Massachusetts, United States

## Abstract

People often wish to simplify their lives as they age, hoping to lead a less complex, stress-free, and happy existence. However, the loss of complexity may actually endanger one’s health. Aging is often associated with the pruning of work, family, and social networks in our external environment, and on a smaller scale, with the degradation of various anatomic structures and physiologic processes internally. This loss of complexity can impair our ability to perform activities of daily life or adapt to surgery or other stressors. We can quantify complexity using measures derived from the concept of fractals that describe patterns of behavior across different scales in space or time. Using these metrics we have shown that complexity loss is potentially reversible. This presentation will describe the measures, mechanisms, and consequences of complexity loss in different human systems, and interventions that can restore complexity and thereby improve functional health in older age.

